# Likelihood-based feature representation learning combined with neighborhood information for predicting circRNA–miRNA associations

**DOI:** 10.1093/bib/bbae020

**Published:** 2024-02-07

**Authors:** Lu-Xiang Guo, Lei Wang, Zhu-Hong You, Chang-Qing Yu, Meng-Lei Hu, Bo-Wei Zhao, Yang Li

**Affiliations:** School of Computer Science and Technology, China University of Mining and Technology, Xuzhou, 221116, China; School of Computer Science and Technology, China University of Mining and Technology, Xuzhou, 221116, China; Big Data and Intelligent Computing Research Center, Guangxi Academy of Sciences, Nanning 530007, China; College of Information Science and Engineering, Zaozhuang University, Shandong 277100, China; School of Computer Science, Northwestern Polytechnical University, Xi’an, 710129, China; College of Information Engineering, Xijing University, Xi’an 710123, China; School of Medicine, Peking University, Beijing, 100091, China; Xinjiang Technical Institute of Physics and Chemistry, Chinese Academy of Sciences, Urumqi 830011, China; School of Computer Science and Information Engineering, Hefei University of Technology, Hefei 230601, China

**Keywords:** circRNA, miRNA, circRNA–miRNA association, convolutional autoencoder, natural language processing, deep neural networks

## Abstract

Connections between circular RNAs (circRNAs) and microRNAs (miRNAs) assume a pivotal position in the onset, evolution, diagnosis and treatment of diseases and tumors. Selecting the most potential circRNA-related miRNAs and taking advantage of them as the biological markers or drug targets could be conducive to dealing with complex human diseases through preventive strategies, diagnostic procedures and therapeutic approaches. Compared to traditional biological experiments, leveraging computational models to integrate diverse biological data in order to infer potential associations proves to be a more efficient and cost-effective approach. This paper developed a model of Convolutional Autoencoder for CircRNA–MiRNA Associations (CA-CMA) prediction. Initially, this model merged the natural language characteristics of the circRNA and miRNA sequence with the features of circRNA–miRNA interactions. Subsequently, it utilized all circRNA–miRNA pairs to construct a molecular association network, which was then fine-tuned by labeled samples to optimize the network parameters. Finally, the prediction outcome is obtained by utilizing the deep neural networks classifier. This model innovatively combines the likelihood objective that preserves the neighborhood through optimization, to learn the continuous feature representation of words and preserve the spatial information of two-dimensional signals. During the process of 5-fold cross-validation, CA-CMA exhibited exceptional performance compared to numerous prior computational approaches, as evidenced by its mean area under the receiver operating characteristic curve of 0.9138 and a minimal SD of 0.0024. Furthermore, recent literature has confirmed the accuracy of 25 out of the top 30 circRNA–miRNA pairs identified with the highest CA-CMA scores during case studies. The results of these experiments highlight the robustness and versatility of our model.

## INTRODUCTION

Over the past few decades, scientists have been dedicated to delving into RNA (ribonucleic acid) and have provided solid evidence that it is an important molecule in animals, plants, microorganisms and viruses [1, 2]. Circular RNA (circRNA) is a fresh long-stranded noncoding RNA (ncRNA) class. CircRNA was not known until 1976 when Sanger *et al*. [3] uncovered viroids as ‘single-stranded and covalently closed circRNA molecules’. In recent years, researchers have found that circRNAs are not only a continuous cycle of covalent closure but also do not have a 3' or 5' polar structure. MicroRNAs (miRNAs) are also a class of single-stranded ncRNAs, which are encoded by endogenous genes. Research has revealed that circRNA is produced by the reversal splicing of mRNA (messenger RNA) precursors (pre-mRNAs) [4] and contains multiple miRNA (microRNA) response elements that can function as miRNA sponges [5]. This implies that circRNA plays a significant role in modulating gene expression and cellular functions in carcinoma [6] and non-cancer diseases [7], especially in cardiovascular diseases. Until now, there have been numerous instances of circRNA–miRNA associations being applied in disease management and drug research [8]. For instance, Wu *et al*. investigated the potential miRNA-binding sites in circRNAs associated with Alzheimer's disease (AD), specifically focusing on miRNAs that are implicated in targeting AD-related genes [9]. Li *et al*. studied the involvement of circRNAs regulates miRNA in kidney disease development and discussed their potential as therapeutic targets [10]. Zhang *et al*. conducted a study on the role of circRNAs in the pathogenesis of autoimmune diseases, highlighting their function as miRNA sponges that regulate various biological processes [11].

Extensive research efforts have been focused on exploring the role of circRNAs have complex associations with miRNAs in various organisms, including animals, plants, microorganisms and viruses. As a result, compelling evidence has emerged to highlight the significance of this molecule. For example, Chen *et al*. [12] have demonstrated that through its sponge activity against miR-30a-3p, circHIPK3 RNA promotes the proliferation and differentiation of chicken muscle cells. Zhao *et al*. [13] found that network of circRNA–miRNA interactions involving thousands of circRNAs and 92 miRNAs to explore the potential functions of plant circRNAs, such as in soybean. Qu *et al*. [14] confirmed that an intronic circRNA has been identified as a novel antagonist against influenza virus, by sequestering a miRNA that targets CREBBP and enhancing the production of IFN-β. Hence, investigating the possible associations between circular RNAs and microRNAs holds promise in enabling biologists to better understand and diagnose the complex mechanisms underlying diseases and ultimately improve the treatment of clinical conditions.

To minimize errors in wet lab experiments, a notable number of computer experiments have been conducted to investigate CMAs [15, 16]. As an illustration, Lan *et al*. [17] introduced a computational approach for forecasting CMAs in NECMA. This method relies on the creation of a heterogenous network using various resources, encompassing the circRNA GIP kernel similarity network, miRNA GIP kernel similarity network and circRNA–miRNA association network. The authors utilized inner product and neighborhood regularization logic matrix decomposition to achieve an AUC of 0.8264 accurate predictions. In addition, Qian *et al*. [18] built a algorithmic model called CMIVGSD, which is based on singular value decomposition and GVAE (graph variational autoencoders), to predict CMAs. The framework was evaluated using 5-fold CV (cross-validation) and attained a prognostication AUC of 0.8804. Furthermore, 6 out of the top 10 predicted CMAs by the CMIVGSD model were validated in PubMed. Guo *et al*. [19] created a computational framework called WSCD, which extracts the attribute feature and behavior feature from multiple sources including circRNA, miRNA sequences and the CMAs network and accurately predicts the CMAs through using Word2vec, SDNE, CNN and DNN. Specifically, the 5-fold CV experiment yielded promising outcomes, with the model achieving an AUC of 0.8898. Furthermore, the top 30 CMAs forecasted by the model were validated through manual retrieval of related literature and databases, thus validating the accuracy and reliability of the predictions. In comparison, Wang *et al*. [20–22] proposed KGDCMI, SGCNCMI and JSNDCMI models, which achieved higher accuracy with prediction AUCs of 0.8930, 0.8942 and 0.9003, respectively. Based on the data collection presented above, it provides a strong basis for predicting CMAs using computer algorithm models.

Although most pre-existing computational prediction methods can excellently estimate CMAs, it still has some limitations. For instance, (1) the previous models exhibit relatively low predicted values and require improvement. (2) The experimental quantity in previous papers is not enough to verify a dependable model.

**Figure 1:**
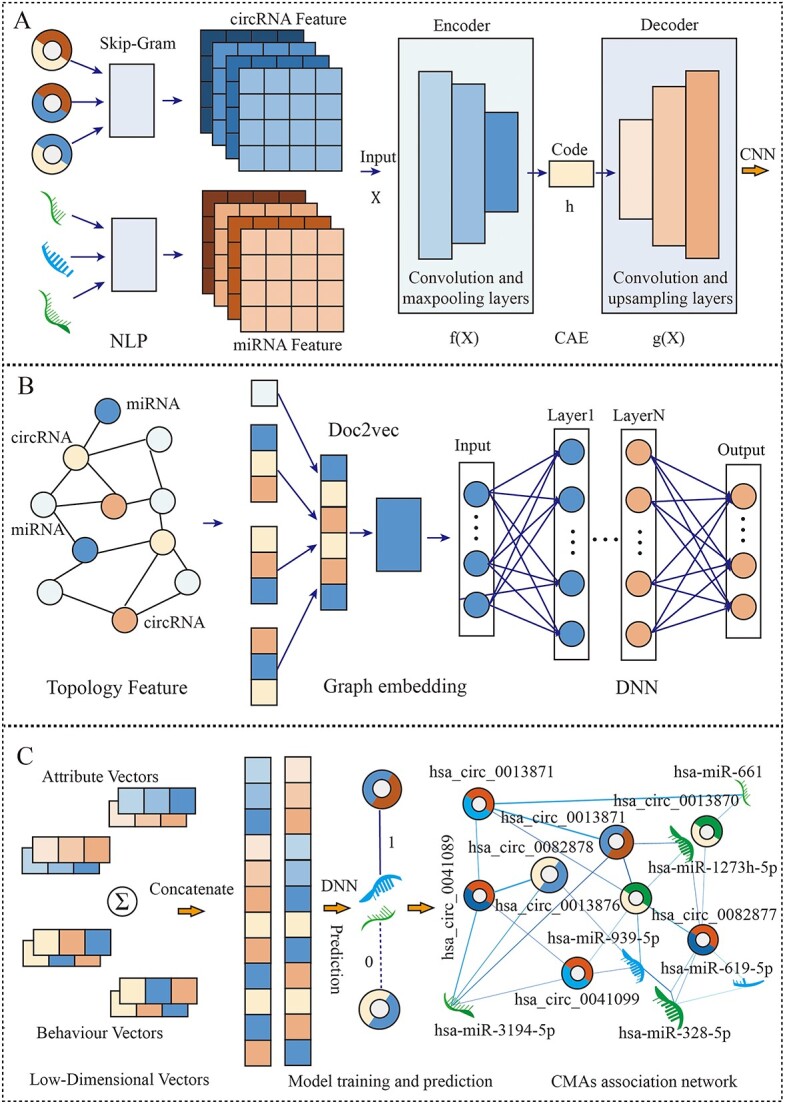
The workflow of the CA-CMA model. (A) ​Extracting low-dimensional biological attribute features vectors. (B) Extracting low-dimensional biological behaviour features vectors. (C) Feature fusion and model learning and forecasting.

After conducting the aforementioned analysis and encouraging advanced field correlation prediction research methods and to address the challenges of time-consuming, labor-intensive and high distortion issues in scientific biological experiments, while simultaneously enhancing the operational efficiency and accuracy of the models, we introduce CA-CMA as a cutting-edge machine learning framework that utilizes text representation learning and neural networks to accurately forecast CMAs. To clarify further, the model can be broken down into three primary components. To predict potential circRNA–miRNA associations, we first combined biological sequence features extracted from convolutional autoencoder–based circRNA–miRNA sequence embeddings and Doc2vec-based validated interaction pairs. Secondly, a dependable molecular association network was constructed using heterogeneous graphs, which were then input into a fusion model of CNN and DNN for low-dimensional embedding vector generation. Finally, it is feasible to use a DNN classifier to successfully infer potential CMAs. To sum up, Figure 1 illustrates the structure of the CA-CMA model, and additional information can be accessed on the following website: https://github.com/look0012/CA-CMA.

## MATERIALS AND METHODS

### Dataset

To ensure a rigorous assessment of the CA-CMA model, our study involved the utilization of three commonly employed datasets in the circRNA–miRNA prediction domain to provide a comprehensive and unbiased assessment of the model's efficacy.

The first data set involved the meticulous clarification of 20 208 CMAs that were experimentally validated and involved 3569 circRNAs and 1152 miRNAs [19, 23]. We refer to this highly curated dataset as CMI-20208. The second data set is called CMI-9589 [23], with 2115 circRNAs and 821 miRNAs. The third data set we are utilizing is CMI-9905 [22], which comprises 9905 experimentally validated CMAs, representing a comprehensive collection of circRNA–miRNA interactions involving 2346 circRNAs and 962 miRNAs. Therefore, we can describe the built dataset that can be seen in Table 1 below and the formula is as follows:


(1) \begin{equation*} D={D}^{+}\cup{D}^{-} \end{equation*}

**Table 1:** Elaborate data regarding CMIs employed in CA-CMA

Dataset	CMI-20208	CMI-9589	CMI-9905
CircRNA	3569	2115	2346
MiRNA	1152	821	962
Interactions	20 208	9589	9905

The sets ${D}^{+}$ and ${D}^{-}$ represent positive and negative samples, correspondingly. Set *D* represents the combination of elements in the dataset. Secondly, DM stores the data in a matrix form, specifically an adjacency matrix. In the adjacency matrix DM, if a connection exists between circRNA $c(j)$ and miRNA $m(i)$, the corresponding $\mathrm{DM}\left(i,j\right)$ is marked as 1; otherwise, it is recorded as 0.

### Feature extraction by Skip-Gram

**Figure 2:**
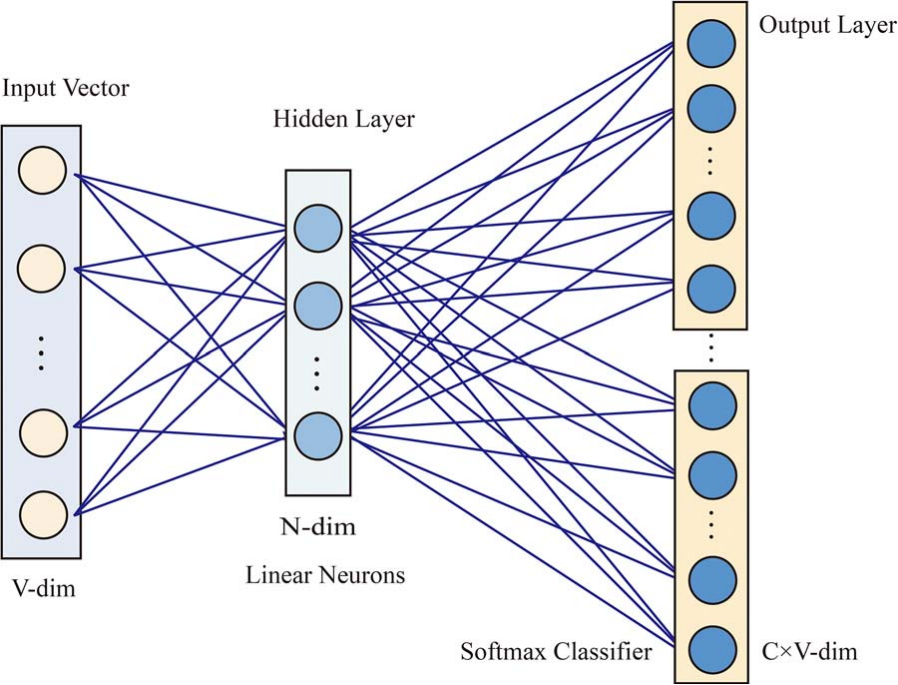
Feature extraction process of the Skip-Gram model.

Word2Vec, initially introduced by Google [24], is a popular word-embedding model [25, 26]. It leverages the relationships between words present in sentences to transform word vectors from a higher-dimensional space to a lower-dimensional space in machine learning. Word2Vec comprises two models, specifically Skip-Gram (we apply this model in this paper) and CBOW. The Skip-Gram model flow is shown in Figure 2. By setting a window size of $k$, the model chooses a central word and regards the other words within the window as background words. The model undergoes training by maximizing the probability of the background word occurring in the proximity of the central word. The training of the Skip-Gram framework focuses on maximizing the occurrence likelihood of background words. More precisely, in the case of a random walk path with length $L$ and a window size of $\omega$, the optimization function for Skip-Gram is defined as


(2) \begin{equation*} \max \left(\frac{1}{L}\sum \limits_{t=1}^L\sum \limits_{-w\le j\le w,j\ne 0}\mathrm{logPr}\left({v}_{t+j}|{v}_t\right)\right) \end{equation*}

Through the utilization of the Skip-Gram model in the gensim python software package [27], we were able to train circRNA and miRNA sequence vectors, resulting in a 64-dimensional objective vector.

### Feature extraction by Doc2vec

The Doc2vec algorithm serves as a document-embedding method, extending the capabilities of word2vec to analyze connections between different documents [28, 29]. The Doc2vec algorithm offers two methods: PV-DM (Paragraph Vector–Distributed Memory) [30] and PV-DBOW (Paragraph Vector–Distributed Bag of Words). While the PV-DM technique resembles the CBOW method of word2Vec in predicting the subsequent word following a series of words and refreshing the paragraph vector, the PV-DBOW technique solely relies on the paragraph vector to predict words that occur in a context, similar to the skip-gram approach. The goal of Doc2Vec is to find the optimum route that optimizes the logarithmic mean probability within a sentence containing $t$ words, as indicated by Equation (4), similar to how Word2Vec operates.


(3) \begin{equation*} \frac{1}{T}{\sum}_{t=k}^{T-k}\log p\left({w}_t|{w}_{t-k},...,{w}_{t+k}\right) \end{equation*}

In other words, the objective of Doc2Vec is to learn the best approach to maximize the probability $P\left({w}_t|{w}_{t-k},...,{w}_{t+k}\right)$, where ${w}_t$ represents the target word when the words ${w}_{t-k},...,{w}_{t+k}$ occur at times $t-k,...,t+k$.

In this research, we employed a vector employing the PV-DM technique to extract features, as suggested by the original study proposing Doc2vec, and the utilization of Doc2vec is accomplished by utilizing 'gensim', a Python package that executes the Doc2vec model.

### Feature extraction by convolutional autoencoder

In contrast to typical AEs, convolutional autoencoders (CAEs) feature weight sharing across all input locations, ensuring spatial locality is maintained [31, 32]. As a result, the reconstruction is accomplished by combining fundamental image patches using the latent code through a linear combination. The CAE architecture draws parallels with the denoising auto-encoder, but with the added aspect of shared weights [33]. The latent representation of the $k$-th feature map is derived from a mono-channel input $x$. The formula is as follows:


(4) \begin{equation*} {h}^k=\sigma \left(x\times{W}^K+{b}^k\right) \end{equation*}

where the entire map receives the bias, incorporating $\sigma$ as the activation function, and the two -dimensional (2D) convolution can be denoted as $\times$.

To promote filter specialization across the entire input, we utilize just one bias per latent map. This decision is rooted in our desire to avoid the introduction of excessive degrees of freedom that could potentially arise from using a bias per element. The reconstruction is achieved through the utilization of


(5) \begin{equation*} y=\sigma \left(\sum \limits_{k\in H}{h}^k\times{\tilde{W}}^K+c\right) \end{equation*}

where a single bias $c$ is assigned to each input channel. $H$ denotes the collection of latent feature maps. The flip operation over both weight dimensions is denoted by $\tilde{W}$. The context governs the 2D convolution described in Equations (4) and (5). The convolution between an $m\times m$ matrix and an $n\times n$ matrix can potentially yield either an ${\left(m+n-1\right)}^2$ matrix (full convolution) or an ${\left(m-n+1\right)}^2$ matrix (valid convolution). The objective function to be minimized is the mean squared error (MSE):


(6) \begin{equation*} E\left(\theta \right)=\frac{1}{2n}\sum \limits_{i=1}^n{\left({x}_i-{y}_i\right)}^2 \end{equation*}

Similar to regular networks, the backpropagation algorithm is employed to calculate the gradient of the error function with respect to the parameters. This can be readily achieved through convolution operations utilizing the following formula:


(7) \begin{equation*} \frac{\partial E\left(\theta \right)}{\partial{W}^k}=x\times \delta{h}^k+{\tilde{h}}^k\times \delta y \end{equation*}

$\delta h$ represents the deltas of the hidden states, while $\delta y$ represents the deltas of the reconstruction. The weights are subsequently updated through the utilization of stochastic gradient descent. This vector was subsequently used to implement a 64 $\times$ 64 target vector using CAE. The flowchart of CAE is shown in Figure 1A.

To compare the feature dimensionality reduction of the CNN model, we propose a PCA (principal component analysis) model, a time-honored statistical technique used for analyzing multivariate data, with its origins dating back to the 19th century and the notable contributions of scientists such as Cauchy and Pearson.

In this study, we extracted low-dimensional feature extraction by PCA through using 'sklearn', a Python package that implements the PCA model. Upon the conclusion of the training process, the obtained outcomes indicate that there are 64 dimensions assigned to each node. Following appropriate parameter tuning, in our study, the model algorithm was executed using PyCharm Community Edition 2021.1 × 64, on a server featuring an Intel(R) Core (TM) i7-12700H CPU and 16GB RAM. In the end, the average duration for training and prediction stages when executing the classifier on a computer is approximately 4 min and 25 s.

## RESULTS

### Evaluation criteria

Different performance indexes, widely used in machine learning, are utilized to assess the prediction efficacy of the proposed CA-CMA model, involving $\mathrm{Spec}.$ (specificity), $\mathrm{Prec}.$ (Precision), $\mathrm{Sens}.$ (Sensitivity), $\mathrm{MCCt}$ (Matthews correlation coefficient) and $\mathrm{Accu}.$ (Accuracy). These evaluation indices are defined as follows:


(8) \begin{equation*} \mathrm{Spec}.=\frac{\mathrm{TN}}{\mathrm{TN}+\mathrm{FP}} \end{equation*}

(9) \begin{equation*} \mathrm{Prec}.=\frac{\mathrm{TP}}{\mathrm{TP}+\mathrm{FP}} \end{equation*}

(10) \begin{equation*} \mathrm{Sens}.=\frac{\mathrm{TP}}{\mathrm{TP}+\mathrm{FN}} \end{equation*}

(11) \begin{equation*} \mathrm{MCCt}=\frac{\mathrm{TP}\times \mathrm{TN}-\mathrm{FP}\times \mathrm{FN}}{\sqrt{\left(\mathrm{TP}+\mathrm{FP}\right)\left(\mathrm{TP}+\mathrm{FN}\right)\left(\mathrm{TN}+\mathrm{FP}\right)\left(\mathrm{TN}+\mathrm{FN}\right)}} \end{equation*}

(12) \begin{equation*} \mathrm{Accu}.=\frac{\mathrm{TP}+\mathrm{TN}}{\mathrm{TP}+\mathrm{TN}+\mathrm{FP}+\mathrm{FN}} \end{equation*}

Here, we employed a robust 5-fold cross-validation ($\mathrm{CV}$) technique to mitigate overfitting and assess the effectiveness of our proposed CA-CMA model. In addition, the evaluation criteria are symbolized by the abbreviations $\mathrm{TP}$ (true positive), $\mathrm{TN}$ (true negative), FP (false positive) and FN (false negative). Furthermore, we generated ROC curves by calculating the true-positive rate (TP rate) and false-positive rate (FP rate) using CA-CMA and computed the average area under the ROC curve (AUC) and area under the precision–recall curve (AUPR) to account for the imbalance.

### Prediction performance of the CA-CMA model

**Figure 3:**
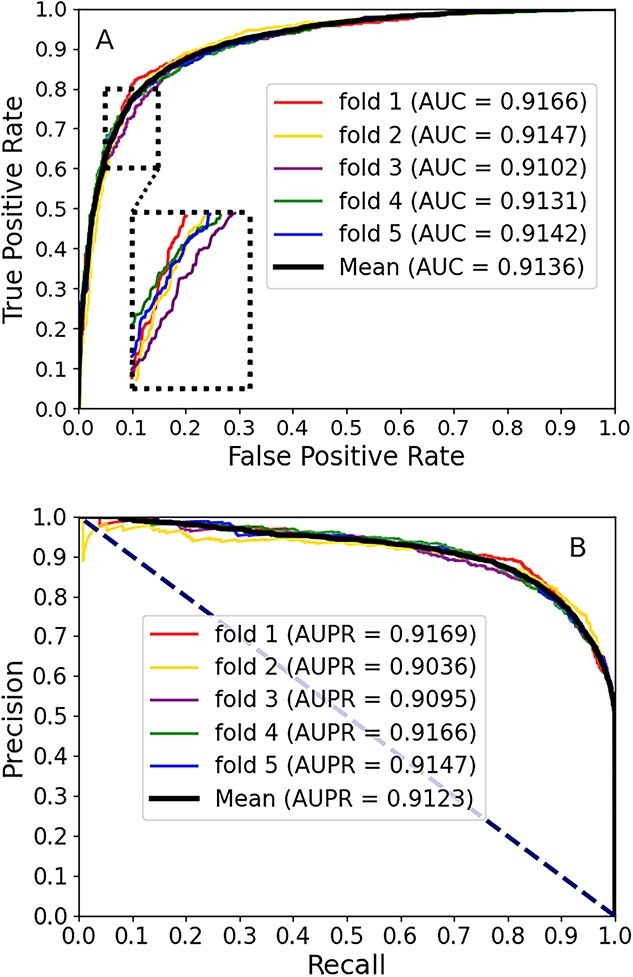
AUC and AUPR were achieved by our proposed CA-CMA. (A) The AUC was obtained by summing the areas under (A) plotting the ROC curve. (B) AUPR refers to the area under the curve enclosed by precision and recall.

By employing the 5-fold CV approach and evaluating the CA-CMA model's performance based on $\mathrm{Accu}.$, $\mathrm{Sens}.$, $\mathrm{Spec}.$, $\mathrm{Prec}.$, $\mathrm{MCCt}$, $\mathrm{AUC}$ and $\mathrm{AUPR}$, we obtained a comprehensive understanding of its efficacy in predicting potential CMAs. Table 2 displays all the experimental outcomes, and the SD and the mean prediction values are highlighted in bold typography, respectively. The AUC values for the 5-fold experiments were 0.9166, 0.9147, 0.9102, 0.9131 and 0.9142, respectively. Therefore, CA-CMA obtained a mean AUC of 0.9138 with an SD of 0.0024. In Figure 3, the AUC can be determined by adding up the regions area beneath the ROC curve depicted in panel A. AUPR, on the other hand, pertains to the region under the PR curve enclosed by precision and recall in panel B, extending to the upper-left and -right corners of the image with a significant AUC, respectively. In summary, the aforementioned statistics demonstrate that the proposed model exhibits cutting-edge performance; it can effectively yield robust evidence for an advanced comprehension of the circRNA–miRNA relationship by accurately forecasting potential CMAs.

**Table 2:** Outcomes of 5-fold CV obtained by CA-CMA

5-Fold	Accu. (%)	Sens. (%)	Spec. (%)	Prec. (%)	MCCt (%)	AUC
Fold1	84.95	86.26	83.64	84.06	69.92	0.9166
Fold2	84.65	87.88	81.41	82.54	69.44	0.9147
Fold3	83.23	81.21	85.25	84.63	66.52	0.9102
Fold4	83.18	84.24	82.12	82.49	66.38	0.9131
Fold5	83.94	85.86	82.02	82.68	67.93	0.9142
Average	83.99	85.09	82.89	83.28	68.04	0.9138
SD	0.810	2.530	1.560	1.000	1.630	0.0024

### Comparison of different feature extraction strategies

**Figure 4:**
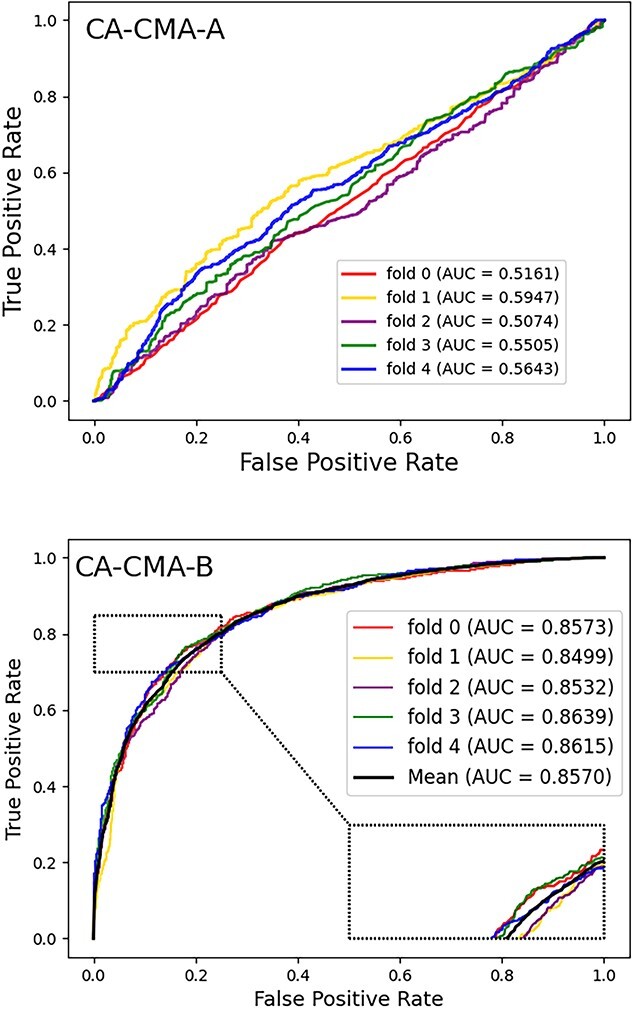
The AUC scores generation of CA-CMA-A and CA-CMA-B.

**Figure 5:**
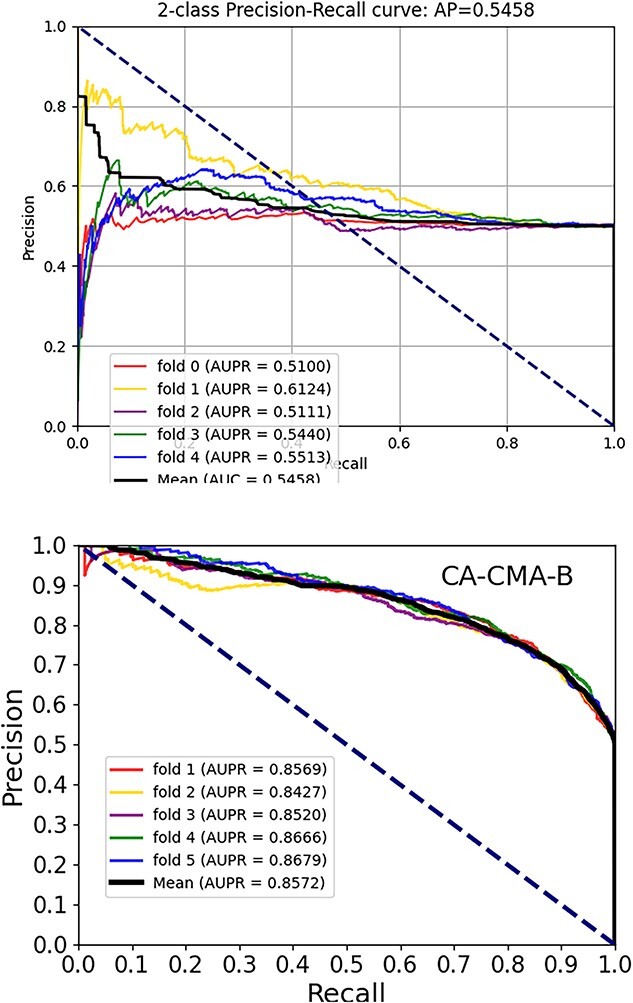
The AUPR scores generation of CA-CMA-A and CA-CMA-B.

Within this section, we pitted the developed framework, which integrates attribute and behavior features, against a couple of cutting-edge models. To be equitable, we employed the two types of features for the purpose of establishing the representation vectors and training the computational model, respectively, while keeping the other aspects of the model consistent. Three approaches are available: ``CA-CMA-A,'' ``CA-CMA-B'' and the amalgamation of the two, referred to as ``CA-CMA.'' The interpretation of the respective parameters is conveyed as attribute-focused (skip-gram, CAE and CNN), behavior-centered (Doc2vec and DNN) and a synthesis of the aforementioned methods, respectively. The depicted outcomes are observable in Figure 4. The results presented in Figure 3 reveal that model ``CA-CMA,'' which incorporates both types of features, achieves a superior prediction AUC of 0.9138 compared to the other models. Furthermore, Figure 5 showcases the benefits of CA-CMA by comparing its prediction AUPR performance with other methods. To sum up, CA-CMA outperforms the two unilateral feature extraction strategies in terms of feature extraction effectiveness.

### Comparison with PCA model

**Figure 6:**
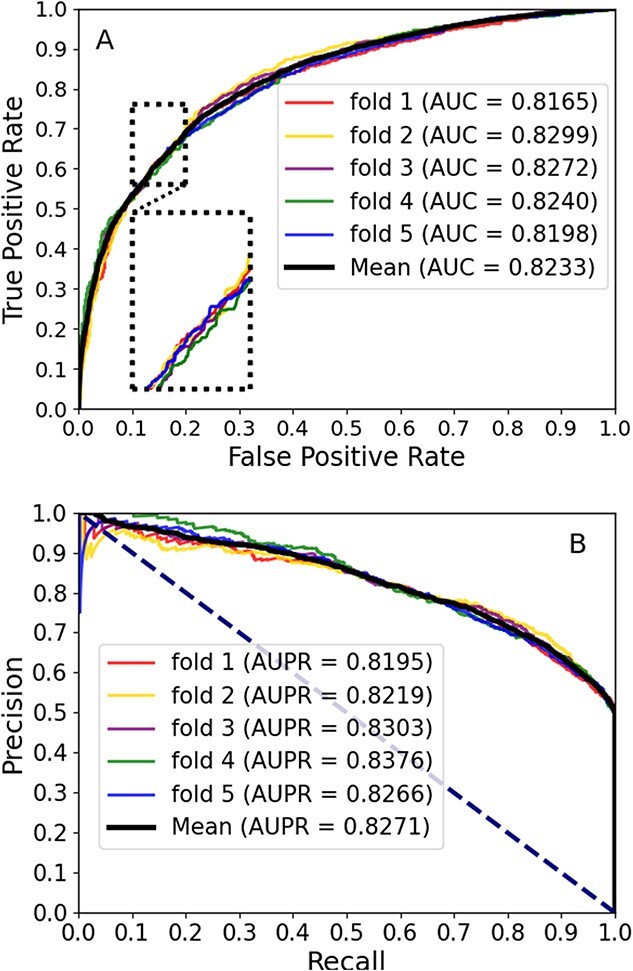
AUC and AUPR were achieved by the PCA method. (A) ROC curves generated by the results of PCA on the dataset using 5-fold CV. (B) AUPR generated by the results of PCA on the dataset using 5-fold CV.

To measure the efficacy of the proposed model, incorporating biological attribute features and multisource semantic information features as model attributes to optimize performance, we conducted a comparison with the low-dimensional feature vector generated using the PCA method. To ensure a fair and consistent approach, we substituted the CNN dimensionality reduction vectors of the CA-CMA model with low-dimensional embedding vectors generated by PCA during the course of the experiment, while leaving the other components of the model unaltered. We utilized the PCA method to train our model using the 5-fold CV technique, yielding the results presented in Table 3, where the bold values represent the mean values obtained by PCA and our model, respectively. As observed in Table 2, CA-CMA exhibited superior performance compared to the PCA model, with its predictions$\mathrm{Accu}.$, $\mathrm{Spec}.$, $\mathrm{Prec}.$, $\mathrm{MCCt}$, $\mathrm{Sens}.$ and AUC exceeding those of the PCA model by 9.27%, 5.05%, 6.92%, 18.5%, 13.49% and 0.0902, respectively. When examining Figures 3 and 6, the superiority of CA-CMA becomes apparent in terms of the AUPR and AUC curve. This result implies that the fusion model, which combines both ``CA-CMA-A'' and ``CA-CMA-B,'' employed by the proposed model is effective in constructing vector characterizations and training the computational model in an effective manner. As a result, it significantly enhances the model and achieves its maximum predictive performance potential.

**Table 3:** Outcomes of 5-fold CV obtained by the PCA model

5-Fold	Accu. (%)	Sens. (%)	Spec. (%)	Prec. (%)	MCCt (%)	AUC
Fold1	74.95	72.73	77.17	76.11	49.95	0.8165
Fold2	76.06	73.74	78.38	77.33	52.18	0.8299
Fold3	74.85	71.92	77.78	76.39	49.78	0.8272
Fold4	73.69	70.71	76.67	75.19	47.46	0.8240
Fold5	74.04	68.89	79.19	76.80	48.34	0.8198
Average	74.72	71.6	77.84	76.36	49.54	0.8235
CA-CMA	83.99	85.09	82.89	83.28	68.04	0.9138

### Comparison with K-Mer model

By comparing the feature vector generated by K-Mer [34] frequencies and probability with the use of CA-CMA as model attributes, we sought to verify whether the performance of the posited model is superior. In order to ensure fairness, we substituted the embedding methods utilized in the experiment with the feature vector derived from K-Mer frequencies and probability, using the same data set in 5-fold CV, while keeping the remaining components of the model unchanged. Table 4 presents a comprehensive breakdown of the comparison results obtained from training the model with K-Mer frequencies and probability, providing a detailed analysis, where the bold values represent the mean values obtained by K-Mer and our model, respectively. As demonstrated in Table 4, the CA-CMA model outperforms the alternative model using the K-Mer model. Specifically, the CA-CMA model achieves higher prediction accuracy, $\mathrm{Accu}.$, $\mathrm{Spec}.$, $\mathrm{Prec}.$, $\mathrm{MCCt}$, $\mathrm{Sens}.$ and $\mathrm{AUC}$ higher than K-Mer model by 22.19%, 20.99%, 21.27%, 44.21%, 23.39% and 0.2442, respectively. The findings reveal that the CA-CMA model is proficient in extracting features, resulting in enhanced model performance and attaining the most competitive outcomes.

**Table 4:** Results of 5-fold CV acquired by K-mer model

5-Fold	Accu. (%)	Sens. (%)	Spec. (%)	Prec. (%)	MCCt (%)	AUC
Fold1	60.40	65.76	55.05	59.4	20.93	0.6523
Fold2	62.42	66.46	58.38	61.50	24.93	0.6780
Fold3	63.28	69.90	56.67	61.73	26.80	0.6851
Fold4	60.76	49.29	72.22	63.96	22.10	0.6625
Fold5	62.12	57.07	67.17	63.48	24.37	0.6699
Average	61.80	61.70	61.90	62.01	23.83	0.6696
CA-CMA	83.99	85.09	82.89	83.28	68.04	0.9138

### Comparison with different classifier models

**Figure 7:**
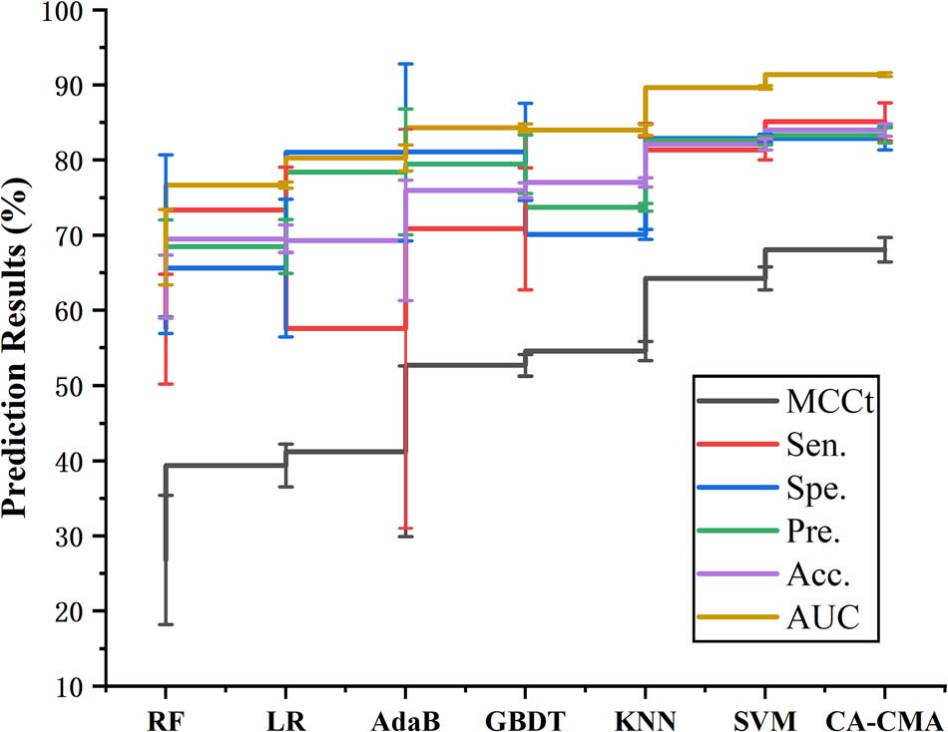
Outcomes of 5-fold CV acquired by different classifier models.

Within this investigation, we assess the influence on the features and performance of CA-CMA by comparing various classifier models, aiming to determine the most suitable classification model. Specifically, we made no alterations to the feature extraction methods while substituting the DNN and CNN fusion model with five distinct classifiers, including KNN (K-nearest neighbor) [35–37], LR (logistic regression) [38, 39], RF (rotation forest) [40–42], SVM (support vector machine) [39, 43], AdaBoost algorithm [44] and GBDT (gradient boosting decision tree) [45–47] for research. Table 5 showcases the average outcomes obtained from the 5-fold CV experiments conducted by the aforementioned models on the identical dataset, and this information is also depicted in Figure 7. Based on Table 5, SVM achieved the second highest ranking in categories $\mathrm{Accu}.$, $\mathrm{Spec}.$, $\mathrm{Prec}.$, $\mathrm{MCCt}$, $\mathrm{Sens}.$ and AUC. However, its results were lower than CA-CMA compared to the best performer. Figure 6 also confirms that the CA-CMA model outperformed all others. To sum up, these findings demonstrate that our CA-CMA model, utilizing the neural network classifier, excels in comparison to other classifier models.

**Table 5:** The average outcomes were obtained using various classifier models

Indicator	AdaBoost	KNN	LR	RF	SVM	GBDT
Acc. (%)	69.290 ± 8.02	77.02 ± 0.62	69.49 ± 1.86	63.140 ± 4.21	82.11 ± 0.77	75.97 ± 0.98
Sen. (%)	57.56 ± 26.55	83.97 ± 0.87	73.35 ± 5.68	57.510 ± 7.29	81.35 ± 1.33	70.83 ± 8.11
Spe. (%)	81.02 ± 11.81	70.07 ± 0.66	65.62 ± 9.18	68.77 ± 11.89	82.87 ± 0.58	81.10 ± 6.48
Pre. (%)	78.40 ± 8.370	73.72 ± 0.52	68.48 ± 3.59	65.580 ± 6.42	82.60 ± 0.58	79.44 ± 3.88
MCCt (%)	41.23 ± 11.34	54.57 ± 1.27	39.39 ± 2.86	26.80 ± 8.610	64.23 ± 1.53	52.66 ± 1.42
AUC	0.8027 ± 0.0175	0.8398 ± 0.0072	0.7667 ± 0.004	0.6837 ± 0.0501	0.8966 ± 0.0024	0.8428 ± 0.0054

### Comparison with other state-of-the-art methods

In recent academic research focused on CMAs, several eminent scholars have put forth diverse methodologies for predicting them. To establish the competitiveness of CA-CMA, we have conducted a comparative analysis of three sets of data alongside the nine most advanced models by the same cross-validation in CMAs prediction. Specifically, we utilize the data sets CMI-9905, CMI-9589 and CMI-20208 as the benchmark for prediction in the CA-CMA model. We are eagerly awaiting the opportunity to compare CA-CMA with the above methods on a level playing field to determine its predictive performance more fairly. Given the nature of the comparison at hand, we have calculated the AUC and AUPR scores generated by previous models and compiled them in Table 6. This table includes our model, as well as a few recently published papers in the emerging field of CMAs prediction with KGDCMI, SGCNCMI, JSNDCMI, BCMCMI [48], DeepCMI [49] and KS-CMI [50]. According to the results presented in the table, CA-CMA achieved the highest AUC and AUPR scores. These scores were significantly superior to those of the second-best KS-CMI model. Besides, as shown in Table 6, it can be observed that the recently published SPBCMI [51] model has a slightly higher AUC than our model by only 0.0005, while the AUPR value is lower than our model by 0.0144. Therefore, considering the comprehensive evaluation, our CA-CMA model outperforms the SPBCMI model. Furthermore, it can be seen from Table 7 that in the data sets CMI-9589 and CMI-20208, the AUC and AUPR values obtained by CA-CMA model are greater than those obtained by other models. Hence, based on the comparison provided above, it can be inferred that CA-CMA offers the most compelling theoretical guidance for future research.

**Table 6:** The AUC and AUPR scores achieved by the various models in the CMI-9905

Methods	KGD CMI	SGCN CMI	JSND CMI	BCM CMI	Deep CMI	KS-CMI	SPB CMI	CA-CMA
AUC	0.8930	0.8942	0.9003	0.9041	0.9054	0.9086	0.9143	0.9138
AUPR	0.8767	0.8887	0.8999	0.8990	0.8978	0.9144	0.8944	0.9088

**Table 7:** Evaluating the performance of various models in CMI-9589 and CMI-20208

Dataset	CMI-9589			CMI-20208	
Methods	NECMA	CMIVGSD	CA-CMA	WSCD	CA-CMA
AUC	0.8264	0.8804	0.9156	0.8898	0.9170
AUPR	0.0048	0.8629	0.9086	0.8847	0.9131

### Case studies

In order to explore the efficacy of CA-CMA in discovering novel miRNA-associated circRNAs, we carried out case studies where the model was trained using established circRNA–miRNA pairs. Subsequently, the trained model was utilized to predict all unidentified CMAs. Unidentified interaction pairs are subsequently ranked based on their higher scores, and their predictive accuracy is validated by cross-referencing relevant research literature or conducting correlation experiments. Among the anticipated outcomes from Table 8, 25 out of the selected top 30 circRNA-related miRNA pairs have been verified and provide a detailed overview of these confirmed pairs. Consequently, these valuable circRNA candidates for miRNA studies are highly likely to be chosen for further experimental investigations in order to mitigate the occurrence of human errors. The findings indicate that CA-CMA exhibits exceptional predictive capabilities when identifying potential CMAs.

**Table 8:** Top 30 CMAs pairs predicted by CA-CMA

Rank	circRNA	miRNA	Evidence	Rank	circRNA	miRNA	Evidence
1	hsa_circ_0013871	hsa-miR-661	Confirmed	16	hsa_circ_0013871	hsa-miR-612	Confirmed
2	hsa_circ_0082878	hsa-miR-661	Confirmed	17	hsa_circ_0082878	hsa-miR-1273 h-5p	Confirmed
3	hsa_circ_0082878	hsa-miR-3194-5p	Confirmed	18	hsa_circ_0082878	hsa-miR-6860	Confirmed
4	hsa_circ_0013871	hsa-miR-4640-5p	Unconfirmed	19	hsa_circ_0013871	hsa-miR-3187-5p	Unconfirmed
5	hsa_circ_0013870	hsa-miR-661	Confirmed	20	hsa_circ_0013870	hsa-miR-612	Confirmed
6	hsa_circ_0082879	hsa-miR-661	Confirmed	21	hsa_circ_0082879	hsa-miR-612	Confirmed
7	hsa_circ_0082878	hsa-miR-665	Confirmed	22	hsa_circ_0082878	hsa-miR-612	Confirmed
8	hsa_circ_0082879	hsa-miR-3194-5p	Confirmed	23	hsa_circ_0082879	hsa-miR-3187-5p	Confirmed
9	hsa_circ_0082878	hsa-miR-939-5p	Confirmed	24	hsa_circ_0082878	hsa-miR-346	Confirmed
10	hsa_circ_0013871	hsa-miR-328-5p	Unconfirmed	25	hsa_circ_0013871	hsa-miR-3187-5p	Confirmed
11	hsa_circ_0082878	hsa-miR-619-5p	Confirmed	26	hsa_circ_0082878	hsa-miR-6860	Confirmed
12	hsa_circ_0013876	hsa-miR-661	Unconfirmed	27	hsa_circ_0013876	hsa-miR-612	Unconfirmed
13	hsa_circ_0082879	hsa-miR-665	Confirmed	28	hsa_circ_0082879	hsa-miR-4739	Confirmed
14	hsa_circ_0082877	hsa-miR-3194-5p	Confirmed	29	hsa_circ_0082877	hsa-miR-4739	Confirmed
15	hsa_circ_0013871	hsa-miR-661	Confirmed	30	hsa_circ_0013871	hsa-miR-6860	Confirmed

## DISCUSSION

The continuous application of high-throughput technology experiments has uncovered the significance of profound genomic data in both understanding comprehensive disease pathogenesis and enabling effective disease prevention, diagnosis and precision medicine. The correlation between circRNA and miRNA undoubtedly holds significant importance in elucidating human pathological processes, unraveling cellular behavior and identifying biomarkers for evaluation and therapy. Bayes formula plays an important role in the exploration of CMA. Bayes' mathematical principle is easy to understand. In simple terms, if you see a person always doing something good, you will infer that a person is more likely to be a good person. That is to say, when you do not know exactly the nature of a thing, you can rely on the number of events related to the particular nature of the thing to determine the probability of its essential properties. In mathematical terms, the more events that occur to support a property, the more likely it is to be true. Unlike other statistical methods, Bayesian methods are based on subjective judgment; you can estimate a value and then revise it based on objective facts.

The advantages of the Bayesian formula include the following: it can make probabilistic inferences in a continually updating dataset, thereby improving the accuracy of predictions. It can handle uncertainty and complexity, as the Bayesian formula can deal with relationships between multiple unknown variables. It can naturally incorporate prior knowledge, combining what is already known with new evidence to arrive at more accurate conclusions. However, the Bayesian formula also has its drawbacks: the choice of prior distribution can have a significant impact on the results. If the prior distribution is incorrectly chosen or estimated, it may lead to bias in subsequent inferences. The Bayesian formula requires the computation of a large number of probabilities, which can result in high computational costs. The Bayesian formula requires modeling all possible outcomes, which can make the model very complex and difficult to interpret.

In this study, the interaction between circRNA and miRNA is also determined using Bayesian methods. Specifically, before determining whether circRNA and miRNA interact with each other, as their characteristics were not previously understood, it seemed that their association could only be randomly judged. However, this is not actually the case. We will utilize the Bayesian method based on accumulated experience to provide clues for judgment. Experience tells us that typically circRNA and miRNA with interactions can exhibit similar characteristics, while those with little correlation show significant differences in features extracted by CMA. Therefore, we often determine whether they interact by observing the attribute and behavioral characteristics of circRNA and miRNA. This is a subjective judgment based on prior knowledge. By constructing models through computer algorithms to predict the interaction more accurately between new circRNA and miRNA, it becomes easier to infer CMA in the future. Therefore, in situations where our understanding of things is incomplete, the Bayesian method is a good way to utilize experience and make more reasonable judgments. It has been well applied to our proposed CA-CMA model. This model effectively combines optimizing likelihood targets in the neighborhood, learning continuous feature representations of words and preserving spatial information of 2D signals. These innovative techniques improve the efficiency and accuracy of the model's predictions.

## CONCLUSION

In order to overcome the time-consuming, labor-intensive and high distortion issues associated with traditional biological experiments, in this research, we propose the CA-CMA framework, an innovative approach for machine learning, enabling the model to extract features comprehensively while effectively preserving the spatial information of a 2D signal. Our framework leverages a fusion model that combines NLP algorithms and neural networks to accurately predict CMAs. Initially, we created a mechanism for generating embedding vectors using word embedding, focusing on the sequence information. We then utilized a CAE and CNN to construct a low-dimensional representation, while still preserving the advantages of strong generalization, eliminating the need for unsupervised data annotation. Similarly, we can obtain paragraph characteristics using Doc2vec. Furthermore, a reliable molecular association network was built by utilizing diverse information, which was subsequently fed into a fusion model consisting of CNN and DNN to predict scores. Multiple evaluation metrics, derived from thorough 5-fold cross-validation experiments, consistently demonstrate the exceptional predictive performance of CA-CMA. Furthermore, our model has undergone six different experiments, each of which has convincingly demonstrated its superiority.

The CA-CMA model showcased remarkable predictive performance in this paper due to numerous advantageous factors outlined below. Firstly, there are no errors or disturbances from wet experiments. Secondly, the feature information is thoroughly taken into consideration. Lastly, a well-optimized model is constructed. Regardless, it is essential to overcome the following restrictions of the CA-CMA model in future studies. (i) Our model merely placed significant emphasis on the direct neighbor information shared among heterogeneous of nodes. (ii) The investigation of extracting features from association relationships is inadequate. Building upon the above discussion and conclusion, our plan is to explore methods to expand the range of aggregated data. This includes the consolidation of information from neighbors located at multiple hops, with the goal of further enhancing prediction performance. Besides, embedding more biological association information and exploring more efficient models are also our focus in the following. Next, we use the prediction of miRNA binding sites to perform CMAs and explore novel feature selection and dimensionality reduction methods to identify the most representative and discriminative features from the vast number of available features. This approach aims to enhance the model's robustness and predictive capabilities.

**Key Points**

- The intricate correlation between circRNA and miRNA serves as a crucial regulatory element in diverse pathological mechanisms and holds significant importance as a molecular player in animals, plants, microorganisms and viruses.
- Our proposition of CA-CMA is grounded in the fusion of CAE and doc2vec, enabling the model to extract features comprehensively while effectively preserving the spatial information of a two-dimensional signal.
- Through seven experiments, the model's robustness and extensive applicability have been fully demonstrated.

## Data Availability

The data can be freely downloaded from https://github.com/look0012/CA-CMA.

## Author Biographies

**Lu-Xiang Guo** is a Master Degree candidate at China University of Mining and Technology. His research interests include bioinformatics, machine learning and complex network.

**Lei Wang** is a Professor of China University of Mining and Technology. His research interests include data mining, machine learning, computational biology and bioinformatics.

**Zhu-Hong You** is a Professor of Northwestern Polytechnical University. His research interests include intelligent information processing and its applications in bioinformatics.

**Chang-Qing Yu** is a Professor of Xijing University. His research interests include neural networks, data analysis and Internet of Things.

**Meng-lei Hu** is a Master Degree candidate at Peking University. Her research interests include the integration of traditional Chinese and Western medicine and machine learning.

**Bo-Wei Zhao** is a PhD student at the University of Chinese Academy of Sciences. His research interests include machine learning, complex networks and graph neural network.

**Yang Li** is a PhD student at Hefei University of Technology, Hefei, China. His current research interests include machine learning and its applications in bioinformatics.
